# Community engagement education in academic health centers, colleges, and universities

**DOI:** 10.1017/cts.2022.424

**Published:** 2022-07-07

**Authors:** Chyke A. Doubeni, David Nelson, Elizabeth Gross Cohn, Electra Paskett, Seleshi Ayalew Asfaw, Mehek Sumar, Syed M. Ahmed, Rhonda McClinton-Brown, Mark L. Wieland, Anita Kinney, Sergio Aguilar-Gaxiola, Lisa G. Rosas, Cecilia M. Patino

**Affiliations:** 1 The Ohio State University Wexner Medical Center, Columbus, OH, USA; 2 Center for Health Equity and Community Engagement Research, Mayo Clinic, Rochester, MN, USA; 3 Center for Clinical and Translational Science, Mayo Clinic, Rochester, MN, USA; 4 Department of Family and Community Medicine, Medical College of Wisconsin, Milwaukee, WI, USA; 5 Hunter College, City University of New York, NY, USA, New York; 6 College of Medicine, and Comprehensive Cancer Center, The Ohio State University, Columbus, OH, USA; 7 Ethiopian Tewahedo Social Services, Columbus, OH, USA; 8 Division of Biological Sciences, University of California San Diego, San Diego, CA, USA; 9 Healthy Communities Branch, Santa Clara County Public Health Department, Santa Clara, CA, USA; 10 Division of Community Internal Medicine, Geriatrics, and Palliative Care, Mayo Clinic, Rochester, MN, USA; 11 Department of Biostatistics and Epidemiology at the School of Public Health and the Rutgers Cancer Institute of New Jersey, New Brunswick, NJ, USA; 12 UC Davis Center for Reducing Health Disparities, Clinical and Translational Science Center, Dept of Internal Medicine, University of California Davis, Sacramento, CA, USA; 13 Department of Epidemiology and Population Health and Department of Medicine, Stanford School of Medicine, Palo Alto, CA, USA; 14 Department of Population and Public Health Sciences, School of Medicine, University of Southern California; Southern California Clinical and Translational Institute, Los Angeles, CA, USA

**Keywords:** Community engagement, education, training, pedagogy, service learning, health equity

## Abstract

Community engagement (CE) is critical for advancing health equity and a key approach for promoting inclusive clinical and translational science. However, it requires a workforce trained to effectively design, implement, and evaluate health promotion and improvement strategies through meaningful collaboration with community members. This paper presents an approach for designing CE curricula for research, education, clinical care, and public health learners. A general pedagogical framework is presented to support curriculum development with the inclusion of community members as facilitators or faculty. The overall goal of the curriculum is envisioned as enabling learners to effectively demonstrate the principles of CE in working with community members on issues of concern to communities to promote health and well-being. We highlight transformations needed for the commonly used critical service-learning model and the importance of faculty well-versed in CE. Courses may include didactics and practicums with well-defined objectives and evaluation components. Because of the importance of building and maintaining relationships in CE, a preparatory phase is recommended prior to experiential learning, which should be guided and designed to include debriefing and reflective learning. Depending on the scope of the course, evaluation should include community perspectives on the experience.

## Introduction

Community engagement (CE) is an ongoing and evolving process of multidirectional collaborations among organizational entities and members of a community. The overall goal is to solve problems and address priorities that matter to a particular community. Meaningful CE is grounded on core principles that promote durable, long-lasting, and equitable relationships among all involved or affected (BOX 1) [1,2]. A key goal of CE is to improve health, broadly defined [3], in the community through clinical, public health, and research programs that are appropriate and culturally aligned with the values and preferences of the population of focus. CE is thus an essential tool for addressing unmet needs in communities that are under-resourced or experience disproportionate rates of preventable morbidity and mortality. CE can help enhance community capabilities for addressing existing or emerging priorities across many areas. However, CE approaches vary in complexity and level of community involvement. These include outreach and consultation that entail low levels of community involvement to community-based participatory research (CBPR [4]) or similarly structured models that enable co-creation and co-leadership with community partners [ 1,5].

BOX 1: Principles of Community Engagement (Adapted from Ref #1)

**Table:** 

Principle	Description
Understand the community	Become knowledgeable about the community’s culture (norms and values), social networks, economic conditions, political and power structures, and demographic trends, and its history and experiences with efforts by outside groups and past engagement activities
Maintain community ownership and control	Remember and accept that collective self-determination is the responsibility and right of all people in a community. Therefore, no external entity should assume it can bestow on a community the power to act in its own self-interest.
Have shared goal or vision for engagement	Be clear about the purposes for engagement or seeking partnership and work collaboratively to have a goal or vision that the community shares
Establish partnership	Establish a partnership with the community to create change and improve health
Be respectful	Recognize and respect the diversity within the community
Build trust	Build and maintain relationships and trust by working with individuals and/or key community leaders or connectors
Mobilize community assets	Identify and mobilize community assets and strengths through developing the community’s capacity and resources to make decisions and take action
Be flexible and adaptable	Recognize that individuals and institutions must be flexible and adaptable to community priorities and changing conditions
Commit to a long-term partnership	Foster community collaboration and understand that community impact requires collective action across sectors in collaboration with members of the community with a long-term commitment among partners
Be trustworthy	Understand that being perceived as trustworthy (individually and organizationally) is essential for sustaining community engagement

The critical role of CE in identifying solutions at intersections among social and structural barriers and health risks and outcomes is well-acknowledged [6,7]. Its increasing recognition is exemplified by the National Institute of Health Community Engagement Alliance (CEAL) against COVID-19 disparities that deployed CE, at scale, across 21 states/territory for pandemic-related prevention, care, and research, including addressing mistrust and countering misinformation [8,9]. CE is a core component of the National Cancer Institute-supported comprehensive cancer centers and the National Center for Advancing Translational Sciences Clinical and Translational Science Award (CTSA) program [10,11]. Although areas of emphasis vary across CE initiatives, they collectively seek to enhance collaborations with communities across the translational science continuum, including clinical care delivery and community-based interventions or programs, to realize meaningful and sustainable community impact [12]. A National Academy of Medicine (NAM) commentary noted, “it is only with community engagement that it is possible to achieve and accelerate progress toward the goal of health equity through transformed systems for health” [2]. CE practice is undergirded by justice, equity, inclusion, respect, trust, community ownership, and long-term commitment (BOX 1) [1]. Trust through relationship building is central to CE, and poor fidelity to CE principles can create or deepen mistrust and hamper efforts to address health inequities, underscoring the need for relational knowledge and lifelong learning as crucial tenets of CE pedagogy.

This article aims to provide a pedagogical framework to support curricula development to improve knowledge and practice of CE across a broad range of learner types, settings, and approaches.

## The Need for a Framework for CE Pedagogy

Although there is growing recognition of the importance of CE [5], today’s workforce needs training to effectively design, implement, and evaluate strategies through meaningful collaboration with members of the community of focus [13]. The goal is for learners to acquire knowledge and skills in CE in research, education, and clinical care or public health practice [14,15]. The knowledge and skills acquired can equip learners to identify exemplars and metrics of success in addressing health disparities [16,17], policy enablers of health equity [18–22], or partner with communities to co-create and share best practices.

CE-related educational content designed ostensibly to improve knowledge, attitudes, and skills exists in many forms in curricula across the educational continuum. However, few published pedagogical frameworks exist to guide CE curriculum development across disciplines. There is also marked heterogeneity across existing content and a lack of a shared approach for CE education [11,23].

### Value of CE Pedagogy for Learners, Communities, and Partnering Organizations

CE pedagogy can help advance health equity by enabling learners to gain deeper knowledge of social determinants of health and gain insights on the societal context of effective strategies to address and reverse the effects of health and social injustices, stereotypes or structural racism, and similar biases [24,25]. Implementing CE curricula thus benefits learners and their careers by equipping them with a deeper understanding of root causes of inequities and their impact on health and wellness. It may also improve the abilities of practitioners to quickly recognize and address emerging community concerns to avoid embedding mistrust in the community. Notably, a well-designed, implemented, and evaluated CE educational program may advance community objectives and reduce the risk of re-traumatization in communities. CE education can also provide community partners or members with opportunities to participate in training, enhance capabilities in communities, and spur community-led action or research by enhancing engagement with academic partners [11,26]. From the perspective of academic institutions and other partnering organizations, CE education can help train a well-prepared workforce for advancing community impact and health equity goals [27]. When designed and implemented strategically through a shared vision, CE curricula informed by a framework as proposed can enable organizations to strengthen their community relationships [28,29].

### Value for Competency-Based Education

Education is accomplished at multiple levels and has traditionally been guided by learning objectives, competencies, and grading standards [30–32]. Undergraduate medical education has moved toward milestones and passing scores. This evolution is also seen in public health education, with graduate training moving to competencies and concepts built around lifelong learning [33]. That evolution dovetails with principles of CE as described earlier [1,2]. CE learning should recognize that one is never entirely "competent" in identifying and working on community priorities. It should be approached as a lifelong process and from a place of humility to promote growth and connectedness to the community. The knowledge and experiences can translate into enhanced skills of co-learning with community partners to advance health equity through activities in education, research, public health programs, and healthcare delivery. Some proposed competencies for CE have been described in the extant literature [11].

### Approach for Informing Recommendations on CE Pedagogy

To support recommendations for curriculum development, we searched the peer-reviewed and grey literature for CE educational programs. Many of the authors, some with community partners who are coauthors of this article, lead CE initiatives in CTSAs and at cancer centers and collectively provided resources and insights with input from community partners. Additionally, we conducted direct outreach to other selected academic centers.

We based recommendations, partly, on educational theories, health equity frameworks, and emerging consensus on principles of CE [1,2,5,13,33–40]. Insights were drawn from a report by an Association of American Medical Colleges (AAMC) panel on public and population health in medical education [14].

### Elements of CE Pedagogy

Learning is iterative and not a linear process, although core cognitive domain content is helpful prior to practical experiences. The proposed curricula framework is amenable to being adapted across a varied set of learners in academic and nonacademic settings. The proposed framework applies to precollegiate, undergraduate and graduate students, faculty, employees engaged in CE, and community members.

Core content on CE delivered using both traditional didactic and experiential methods should be considered an essential component of curricula at all levels of clinical and public health training and practice and related fields [13]. Core competencies should include: 1) social determinants of health and historical injustices; 2) CE principles and community knowledge and relationships; 3) resource sharing and communication; 4) personal traits for successful CE, including cultural humility; 5) elements and value of community-engaged research; 6) program evaluation for CE; and 7) dissemination and advocacy in CE [11]. The importance of sustainability and trustworthiness should be interwoven throughout the curriculum [2,26,41,42].

Some of the existing CE-related educational content [12,14,43–45] focus on specific activities or models (e.g., CBPR) [13,15,46,47], and others on service-learning (or critical service-learning), which is the predominant form of pedagogy in academic health institutions [36,43,48–52]. As discussed later, fidelity to CE principles in service-learning pedagogy is not explicit. It thus may not be suitable as the sole source of community-engaged education in its current form.

Several essential elements to CE pedagogy can be drawn from the existing literature. First, the core set of CE principles should be integral to curricula design [1,2]. Second, because CE is an ongoing and dynamic process, a lifelong learning approach is needed. Third, effective CE strategies require enhanced relational knowledge and emotional intelligence for successful relationship building that promotes respect, trust, and prioritization of community ownership over individual exigencies. Fourth, community members should be involved in designing the course and included as faculty, which can increase the focus of training programs on community values and priorities and community-identified strategies [26].

Thus, it is essential to have clearly defined roles for community members, either as teachers or facilitators, and not simply involve them as "subjects" of the educational program to promote co-learning with community partners [26]. This bidirectional design enables the development of a shared vision in community-engaged programs and reinforce the construct of community ownership, which should encompass dissemination of products of research or other initiatives through peer-reviewed publications or conference presentations. Learners must understand the importance of timely return of results to the community and the inclusion of community members both in interpreting the findings and as coauthors.

Overall, CE pedagogy should have: 1) a preparatory phase; and 2) experiential learning such as observerships or supervised activities. An example of that approach is the PARE (Preparation, Action, Reflection, Evaluation) model (Table 1) [43]. Similarly, Kolb’s Experiential Learning Theory proposes four stages: concrete experience, reflective observation, abstract conceptualization, and active experimentation [53,54]. Thus, the design should incorporate adult learning principles, including experience, mentorship, self-direction, and self-motivation [33,35]. Consequently, learning can be self-directed through online modules, individually or in groups, using in-person or virtual platforms. Experience suggests that in-person training and interactions may foster greater community involvement and reinforce relational and reflective skills. We recommend using Universal Design for Learning principles to help make the experience inclusive and accessible for all learners [55].

**Table 1.** Modified PARE (Preparation, Action, Reflection, Evaluation) Curricula Planning Approach

PARE Model(43)	Approaches	Description
**Preparation**	Classroom didactics or self-directed learning, and case-based learning	Learner assessment, including knowledge of pre-requisites (see Box 2) Define principles of community engagement Describe community engagement approaches or strategies, distinguishing between service and co-learning, and describe components, including community advisory boards and their roles and limitations in community engagement Community assessment processes and tools and stakeholder and asset mapping processes Understand community-engaged research. Describe the benefits of community engagement for learners, institutions or organizations, and community Describe outcome measures for community engagement and identify measures of community impact Describe good practices, benefits, harms, and pitfalls of community engagement Identify and use case studies, reflective exercises and facilitated discussions Describe key organizational capabilities needed for community engagement
**Action** Requires faculty/mentor versed in the principles and practice of community engagement and community-engaged research	Discipline-based model	Learners have a presence in the community throughout the learning period
	Problem-based model	Learner with faculty mentor does a community assessment to understand community and priorities
	Capstone course model	In education programs, perform a synthesis of learner’s knowledge, typically during the program’s terminal year (i.e., master’s program). This should draw on the cumulative course work and community activities.
	Community engagement internship model	Learner engages in a designated community during dedicated times of the week. Focus on learning how to enhance community capability with an expectation that both the community and learner benefit.
	Community-engaged research model	Learn community engaged research approaches and toolkits on how to collaborate with community in research. Learn inclusive research practices such as inclusive participation in clinical studies.
**Reflection**	Regularly reflect on experiences	Create self-awareness by drawing on learning objectives and guided by relevant theoretical, methodological, and practical applications
**Evaluation**	Modified New World Kirkpatrick Model(14)	Evaluate learners, faculty and community members on the experiences in domains of Reaction, Learning, Behavior or application, Results, and Systems-based outcomes

## Curricula Design Considerations

### Learners and Faculty Considerations

CE learners vary from precollegiate to faculty, community members, and employees of community-based organizations and private and public agencies. As described earlier, learners acquire CE skills through experiential programs working with community members to understand co-learning and co-creation and community assessment and prioritization. Thus, educators need to have a strong foundation in CE principles and practice, which implies that curricula implementation may begin with ‘training the trainer [11].’ Ideally, educators should have deep knowledge of the community and have community relationships to guide learners and avoid deepening of mistrust during experiential learning [45].

### Curricula Resources

There are many resources about CE for learners [6,14]. An authoritative text on CE principles, first published by practitioners in the field in 1997 and updated in 2011, is essential reading for learners and practitioners alike [1]. NAM publications on CE can also provide an excellent background for learners [2]. Other resources include examples of community-engaged research projects [12,56–59], narratives about pitfalls in CE [4], toolkits for community-academic or healthcare-public health collaborations [60–62], materials on community health indicators [40,63], social-ecological model [37], and frameworks on health equity [39], social determinants of health [8,38,64], and program evaluation [65]. A 1938 lecture by Stampar on rural health provides an excellent overview of reasons and benefits of CE and the interconnectedness among social, economic, and health inequities [66]. The Centers for Disease Control and Prevention (CDC) hosts many publicly available community-level data resources such as PLACES [67], which is derived from Behavioral Risk Factor Surveillance System data [68,69]. Mature CBPR programs, including the Rochester Healthy Community Partnership [57,70] and the FAITH! (Fostering African American Improvement in Total Health) programs at the Mayo Clinic [59,71,72], can provide a solid and authentic model of CE experiences.

Critical service learning is a widely used nontraditional pedagogy of experiential learning through involvement in community-oriented activities with varying levels of structured experiences and reflections [36,43,48–52]. While not synonymous with CE, some elements of critical service-learning are consistent with the intent of community-engaged education [15,52]. The service-learning model carries a community deficit framing (service learning) and infers potentially inaccurate connotations that learners deliver services desired or needed by the community. It can thus potentially perpetuate “town and gown” perceptions among community members. Instead, CE education should emphasize humility and apply core principles of bidirectionality with an emphasis on understanding community assets and ownership and, in the context of community-engaged education, community members as teachers. Thus, the structure of critical service learning should be reconceptualized to integrate didactics on CE principles with examples of the intended purpose of CE and outcomes before the community experience, which in turn should be followed by an evaluation that emphasizes lessons learned rather than services learners purported provided.

### Curricula Components

Ideally, CE training should be required for all learners and for employees in organizations seeking to collaborate with communities. The content and activities should be designed and implemented collaboratively with community partners to reflect local needs and assets [11,23]. The structure should encompass didactics, experiential learning or “field” work, personal reflection, and educational outcomes assessment incorporating community member perspectives [11]. Modules could be posted online and on institutional learning platforms for new and existing employees and set as required training with regular refreshers. Content could be provided online for community members and as part of orienting community advisory board members along with regular refresher training.

An abridged curriculum of a course taught at Stanford is provided in Appendix A. The course, co-developed and co-taught with community partners, includes facilitated discussions and peer-to-peer feedback to enhance co-learning among learners and community members. It also includes content on building self-awareness, and addresses competencies in professionalism and communication skills. Other examples are courses on community-engaged research hosted by the Tufts CTSA program [73] and by the Detroit Urban Research Center (DURC) with the University of Michigan [47]. The course materials provide practical examples of forming and working in community partnerships, including Memoranda of Understanding or Agreement. The Agency for Toxic Substances and Disease Registry (ATSDR) of the US Department of Health and Human Services has several online modules with course objectives and videos that are easily accessible to learners and faculty [12]. The AAMC Expert Panel on Public and Population Health in Medical Education report provides an example of a related curriculum [14], including a detailed evaluation framework based on the New World Kirkpatrick Model [74].

The Tufts CTSA course “Building Your Capacity: Advancing Research through Community Engagement” aims to help communities increase their capacity to participate in research efforts [73]. The DURC online course entitled “Community-Based Participatory Research: A Partnership Approach for Public Health” [47] focuses on researchers, “health and human service practitioners,” and members of community-based organizations. The DURC learning objectives encompass 1) rationale, definition, and core principles of CBPR; 2) strategies for forming, maintaining, sustaining, and evaluating CBPR partnerships; 3) data collection methods and interpretation; 4) methods for dissemination and translation of research findings; and 5) benefits and challenges for using CBPR for research and social change [47].

The approach and examples described above aim to provide faculty with the tools to develop the goal, learning objectives, and evaluation of learning outcomes (Table 1). For example, faculty could frame the overall goal of a CE curriculum as aiming to enable learners to effectively demonstrate the principles of CE in working with community members. The course can be divided into several modules preceded by learner assessment as described in the following sections. The content and context should guide the length of the training and whether it is a standalone course or integrated in related courses.

### Learner Assessment

Learner assessment is essential to inform curricula adaptations to unique needs or areas of emphasis [75,76]. Individuals self-evaluate their current level of knowledge and experience to ascertain prior knowledge, experience, readiness, and capacity to embark on CE. Prior to practicums, it should be clear to the community who the learners are, goal, and how much time they are expected to devote to a particular project or community. This transparency can help clarify expectations and avoid potential disappointment and resentment from all parties involved, and ensures that the relationships last well beyond the project or any one individual [45]. Although many communities welcome students and academic members, mistrust occurs or deepens when well-intentioned people at all levels overpromise and underdeliver on commitments. Additionally, creating narratives that are potentially stigmatizing for the community or mischaracterizing observations in the community can undermine trust building.

### Course Pre-requisites

Prerequisites cover areas from many disciplines and should be tailored to local needs, such as rural or urban communities or a community of people with specific conditions such as substance use disorder. In general, prerequisites should cover core content related to health and wellness in communities (Box 2). The topics include overviews of essential functions of public health; health disparities and definitions of critical constructs involved, including various stratifications; community health indicators; social determinants of health or vital conditions of health frameworks along with social causation of disease; the history of social, health, and research injustices, including structural racism and intersectionality; and applicable health and behavioral theories specifically social-ecological model [8,37–40,63,64]. The prerequisites may be built into required readings or integrated into the core contents.

BOX 2: Course Pre-Requisites

- Essential functions of public health;
- Health disparities (including various stratifications) and definitions of key constructs involved;
- Community health indicators;
- Review of health inequities and social injustices – be able to describe the history of social, health, and research injustices in communities, including structural racism and intersectionality;
- Review the historical basis of mistrust and distrust;
- Social determinants of health or vital conditions of health framework along with social causation of disease, including linkage with biological mechanisms; and
- Relevant health and behavioral theories specifically social-ecological model

### Didactic Component

The didactic component should cover the fundamental principles of CE with applications in research, clinical care, public health, and educational programs [4,45]. It should include community assessments and lessons learned on CE to highlight both benefits and potential harms or pitfalls as exemplified by the DURC course [47,77,78]. It should draw on core competencies to include content on understanding the role and limitations of community advisory boards and how the role of community members should be honored on CE projects. Content should also include understanding the organizational capabilities or toolkits needed for CE [1,12]. The time horizon for meaningful results and the importance of returning research results should be included.

Emphasis should be placed on understanding that prioritization should be done by or with the community and that the meaningfulness of outcomes is determined primarily from the community’s perspective. Locally relevant case studies, similar to those offered by ATSDR, should illustrate creating shared vision and outcome metrics, pitfalls and lessons learned, and how challenges may be overcome; this is particularly important if a practicum or field experience is part of the training [12].

### Practicums

Didactics provide a foundation but should never be considered sufficient because CE does not happen from reading a paper or book, or from behind a computer [13]. Facilitated experiences through practicums are essential for development and maintenance of CE skills. When done stepwise, CE practicums and experiences provide a guided intersection point for the learner with the community [45].

Guided practicums enable learners to understand the community, learn from the community and understand the process of bidirectionality in acting on an issue (Box 1) [1,2]. An orientation session allows discussion about learning objectives and framing of the experience, and to have expectations laid out before the practicum and revisited during regular meetings between faculty and learners. Expectations on self-directed learning and availability for the program are crucial to a successful experience. A progress report generated around the middle of the program can allow discussion of the trajectory and potential adjustments. At the end of the program, an exit interview can assess accomplishments.

We emphasize that students embark on practicums only when both the student and the faculty (ideally with community member input) believe that the student is ready to interact with the community. This approach should be clarified and mutually agreed upon with the learners from the outset as part of the course orientation. The rationale is that sending students out to the community unprepared and unsupervised can jeopardize partnerships.

To teach the skills of seeking and incorporating community feedback and the return of results, we recommend that learners present their experiences or findings to the community members and/or community advisory boards and work with mentors to provide a final report back to the community involved in the learning experience.

### Reflective Learning

Reflective exercises and facilitated discussions are essential to underscore the critical role of relationships in CE. Contemporaneously with structured didactics and practicums, self-awareness and reflective exercises help improve knowledge and skills and address misperceptions and even challenges related to CE. They reinforce the synergism across learning, action, and reflection [79]. Reflective learning exercises can adapt approaches used for understanding and teaching emotional intelligence, particularly those related to social change [34,80–80]. Keeping a reflective journal or field note during the experience is an excellent way to record thoughts and feelings about things that went well and those that did not go well or as expected. A formal debrief should be done in a safe environment focusing on lessons learned – what worked and what did not work and adaptations that were made. While this can be done as a one-on-one or self-guided exercise, it should ideally be conducted as a group exercise to share the lessons learned among learners.

### Evaluation and Assessment Tools

Evaluation with formative and summative assessment should occur at various stages of the learning process to allow adjustments to be made [83]. The course and learner experience should be evaluated by the learner, the facilitator, or the teacher with CE experience and community members [45]. The evaluation framework of the AAMC Expert Panel is a good model for all participants in the course, including community members and faculty leads or facilitators (Table 2) [14]. A sample of evaluation areas is provided in Table 3, and an example is provided in the Tufts CTSA course [73].

**Table 2.** Examples from the Modified New World Kirkpatrick Evaluation Model

	Modified Kirkpatrick levels				
	Reaction	Learning	Behavior or application	Results	Systems-based outcomes^3^
Description/application (varies across learner types^1^)	Find it favorable, engaging, relevant for career, and beneficial to communities	Acquire knowledge, skills, attitudes, confidence, and commitment to CE	Apply the knowledge in their careers or practices	Lead to specified outcomes	Lead to systemic change in institutions and the community
Evaluation methods	Survey of learner and community partner perceptions^2^	Multiple choice questions test, reflections on attitudes or beliefs, confidence in addressing CE practice	Gain CE competencies; Applying CE skills in community activities or formal advocacy	Dual degrees, workforce focused on prioritized areas of need, infrastructure within the institution	Community health improvement, meeting Community Health Needs Assessment objectives, or has policies addressing gaps/needs
Outcome metrics in CE education for learners and community	Positive experiences, identifying opportunities to collaborate, endorsement of a collaborative environment, interest in ongoing participation in CE programs	Knowledge, attitudes, and confidence in CE; principles of CE; asset/needs assessment and prioritization; identified sources of health inequities and how to address them; how to integrate CE into research, education, and clinical care; commitment to community-academic collaboration	Apply CE skills and lessons in educational, clinical, and research settings and health advocacy such as CEnR or involvement in community-led research or outreach that are aligned with community priorities	Program influences learner career choice Number of faculty involved in CE and intellectual resourcesCE advocacy and policy initiatives	Improvement in community prioritized areas and outcomes Community priorities guide educational activities to achieve local health goals Policies (institutional, local, state, and national) are implemented to improve community health Workforce trained for community prioritized areas

Abbreviations: CE = community engagement; CEnR=Community-Engaged Research.

^1^Students, faculty, and community members who are learners of the CE education.

^2^Tools recommended include Center for Health Care Strategies, Inc.; Partnership Assessment Tool for Health; National Collaborating Centre for Methods and Tools Partnership Self-Assessment Tool; and The CDC’s Evaluation Guide: Fundamentals of Evaluating Partnerships [14].

^3^Some examples of community health indicators can be found in the Agency for Toxic Substances and Disease Registry (ATSDR) website including the Action Model [12].

**Table 3.** Sample evaluation components

Learning Objective	Evaluation
Define community and community engagement	Class discussion. Each student provides a definition of community and community engagement
Describe principles of community engagement	Submit a written report applying the principles with a health or social justice issue; Use a quiz or multiple choice question format
Describe the types of community engagement	Work in a small group and do a class presentation: select a health or social issue and provide examples about how the issue can be addressed with different types of community engagement approaches
Explain the historical basis and best practices for community engagement	Select a community that is underserved with health services and write a report explaining why and how the past is important and apply best practices for community engagement
Explain how community engagement may address historical trauma	Submit a report on how community engagement may address historical trauma in the selected community
Describe the importance of equity and inclusion in community engagement work and socio-cultural difference considerations	Class discussion. Each student describes the importance and opportunities and challenges related to socio-cultural differences and related historical basis.
Identify priorities, preferences, and needs through the lenses of power, privilege, and social justice with a community of focus	Conduct a community needs assessment on a focused community; identify and survey key stakeholders, create a summary report, and present and discuss the findings
Identify the potential impacts of community engagement for a community	Class discussion about the potential impact of community engagement on the selected focus community
Describe how community engagement advances clinical and translational science	Submit an evaluation essay about how community engagement within the context of the research plan can advance clinical and translational science and ideals of inclusive research
Develop community partnerships and describe strategies for maintaining them (advanced)	Work in small group to create a written report describing the types of partners, the rationale for selecting them, and processes for building and maintaining them. Include testimonials from at least 5 partners.
Develop a research plan with the community (advanced)	Work in small groups to create a research plan (or community action project) describing the processes for engaging the community along the research continuum from formulating study questions and hypotheses, intervention design, implementation, analysis, dissemination of findings, and next steps.
Reflective exercise	Learners log in a journal during practicum and submit to the faculty mid-term and the last class; may include a group reflective exercise

## Conclusion

CE education is essential for addressing health, wellness, and inequities, particularly in under-resourced communities. It should be considered an essential component of training at all levels of education because it can help create a workforce equipped to address societal and structural barriers, including the adverse social factors, that impede progress towards health equity. The training should encompass understanding and applying core CE principles to promote equal partnership and collaboration, introspection and self-awareness, and practicums to ground the experience. Curricula should be designed with community partners participating as teachers or facilitators and not just as “subjects.” CE practitioners who have not been trained in CE could benefit from a course in the fundamentals, reinforcing that communities are constantly changing and CE education is a lifelong learning process. Institutional adoption and investment in CE education as an enduring learning process can enhance the workforce and strengthen community relationships and impact.

## Supplementary material

For supplementary material accompanying this paper visit https://doi.org/10.1017/cts.2022.424. click here to view supplementary material
