# Supplementary material for: Community engagement education in academic health centers, colleges, and universities
Source: J Clin Transl Sci. 2022 Jul 7;6(1):e109. doi: 10.1017/cts.2022.424 (PMC9549477; doi:10.1017/cts.2022.424)
Supplement: Supplementary file 1 [file S2059866122004241sup001.docx]

**Supplementary Materials:**

**Appendix A**: Course on “The Science of Community Engagement Community Engagement in Health Research”

**Brief course description:**

This course focuses on community engagement principles that can be applied to diverse health-related research topics and stages of research along the continuum of translational science. The goal is to teach students how to incorporate community engagement into all phases of research including definition of the research question, study design, implementation of the research, data analysis, and dissemination of findings.

**Course objectives:**

1. Describe the value of incorporating community engagement into translational science to improve the research process, outcomes of research, and ultimately to improve health.
2. Articulate benefits, challenges, and solutions for community engagement specific for each phase of research, including choosing a research question, designing the study, identifying measures and outcomes, implementing the study, analyzing data, and disseminating findings.
3. Examine one’s own identity, biases, and stereotypes and how they may influence the approach to community engagement and health research.
4. Design effective and comprehensive plans for engaging relevant communities/patients/stakeholders in each phase of translational research from identification of the research topic to dissemination of
5. research findings.
6. Identify relevant ethical principles in community-engaged research designs and develop strategies to overcome ethical concerns.
7. Apply community engagement approaches that can be tailored for specific research topics, communities or stakeholder groups, and phases of translational research.
8. Develop strategies for evaluating engagement efforts including identifying metrics and using both qualitative and quantitative methodologies.

**Involvement of community partners:**

Small groups of students are paired with a community partner throughout the course with the goal of designing a research study that reflects the needs and strengths of the partner. This project is broken down into weekly milestones:

1. Problem definition
2. Logic model or theory of change
3. Research question
4. Research design
5. Engagement plan
6. Engagement evaluation plan

Ideal community partners for this course are those with longstanding relationships with the instructor and/or institution. Partners participate throughout the course including in evaluating the final project. Partners are paid an honorarium for their time.

**Assignments:**

Assignments are designed to provide students with community engagement skills:

1. Role of identity in community engagement: Self-reflection activities
2. Partnership agreements: Develop a written agreement between a researcher and community partner
3. Each-one-teach-one: Learn about an engagement strategy and teach fellow students
4. Compare and contrast: Re-design a study that was not conducted with community engagement principles
5. Develop a community advisory board plan: Design the recruitment, engagement, and evaluation strategy for a community advisory board

**Developed by:**

Lisa Goldman Rosas, PhD MPH: Assistant Professor, Department of Epidemiology and Population Health, Stanford University

Rhonda McClinton-Brown, MPH, Director, Healthy Communities Branch, Santa Clara County Public Health Department

Patricia Rodriquez Espinosa, PhD MPH, Instructor, Department of Epidemiology and Population Health, Stanford University
